# Bilateral Optic Neuritis Associated with the Use of Infliximab

**DOI:** 10.1155/2011/232986

**Published:** 2011-11-20

**Authors:** Jan O. Aasly

**Affiliations:** ^1^Department of Neurology, St. Olavs Hospital, 7006 Trondheim, Norway; ^2^Department of Neuroscience, Norwegian University of Science and Technology, 7489 Trondheim, Norway

## Abstract

A 40 year old man was admitted with a 2 weeks history of headache, blurred vision and bilateral optic neuritis. During the 6 months period prior to admission he had treated with infliximab infusions for prsoriasis arthritis. He had 0.2 vision in right eye and 0.5 in left Fundoscopy showed moderate disc swelling more on the right than on the left side and right-sided splinter heamorrhages at the disc margin. The intracranial pressure was normal. He was treated with oral methylprednisolone, 100 mg daily for 1 week. His vision improved gradually and when seen 10 weeks later his visual acuity was 1.0 in both eyes and he had normal visual fields. Optic neuritis is a rare but well recognized serious adverse effect of treatments with tumor necrosis factor (TNF) antagonists. This case report illustrates a rare but typical side effect of a TNF alpha inhibitors used for treating a number of inflammatory diseases. These reactions usually appear during first year of treatments and never after the first one or two infusions. Both genders and all ages are affected. In some patients the visual defects are irreversible.

## 1. Introduction

Headache and bilateral visual impairment in young adults without obvious MRI abnormalities may represent a diagnostic challenge for several medical specialities. Optic neuritis is a rare but well-recognized serious adverse effect of treatments with tumor necrosis factor (TNF) antagonists [1–3]. This case report illustrates a rare but typical side effect of a TNF alpha inhibitors used for treating a number of inflammatory diseases. 

## 2. Case Report

A 40-year-old man was admitted to our outpatient clinic at the Department of Neurology with a 2-week history of headache, blurred vision, and bilateral optic neuritis. He had been of good health except for psoriasis mainly of the plaquelike type and psoriasis-associated arthritis. His psoriasis had been treated with topical agents and was well controlled. The psoriatic spondylitis responded less well to anti-inflammatory therapy. 6 months before admission he had also been treated with infliximab infusions. Infliximab infusions reduced his arthritis considerably with less pain and improved functional level. The fifth infusion of treatment had been given 37 days prior to admission. He was not on any other medication.

A few days before admission he had noticed headache, narrowing of his visual fields, and blurred vision. On admission to hospital he had 0.2 vision in right eye and 0.5 in left eye with abnormal visual fields ad modum Donders. Fundoscopy showed moderate disc swelling more on the right than on the left side. He had right-sided splinter heamorrhages at the disc margin. Fluorescein angiography showed vascular leakage bilaterally, more on the right optic nerve head than on the left. 

A lumbar puncture was performed and the intracranial pressure was 20 cm H_2_O which is within the normal range. There was normal CSF cell count and no signs of intrathecal immunoglobulin productions. A brain MRI showed hyperdense signals in both optic nerves, more on the right than on the left side. There was no hyperdense lesions or demyelination in the CNS. He was treated with oral methylprednisolone, 100 mg per day for 1 week and then tapered off. His vision improved gradually, and when seen 10 weeks later his visual acuity was 1.0 in both eyes and he had normal visual fields.

**Comments**

There are several TNF antagonists on the market and optic neuritis has been reported after treatments with adalibmumab, infliximab, and etanercept [4]. Although there might be TNF antagonists available so far not being connected to this type of adverse effects, our patient declined any further treatment. This was probably a very wise decision. Optic neuritis is probably related to this type of drugs and switching from one TNF antagonists to another could probably induce the same type of reaction.

Our patient recovered completely within 6-7 weeks after onset of symptoms and about 2 months after last TNF antagonist infusion. In most patients vision seems to improve but not all patients recover completely. In a minority of cases, no improvements have been reported [1]. In nearly all reports, IV steroids or oral steroids have been given [4]. Typically, these reactions appear during first year of treatments and never after the first one or two infusions. Both genders and all ages are affected.

TNF antagonist have been used in several inflammatory diseases, and optic neuritis has been reported in patients with rheumatoid arthritis, psoriatic arthritis, inflammatory bowel diseases, and bilateral anterior uveitis [4–6].

In most cases with TNF-antagonist-induced optic neuritis brain, MRI shows enhancements of the optic nerves but rarely demyelinating lesions in the CNS. There is no known correlation between TNF antagonist treatment and multiple sclerosis, MS. There might be a higher rate of demyelination among patients with inflammatory diseases compared to controls. This might only reflect the underlying predisposition associated to their autoimmune disease. Most TNF-antagonist-induced optic neuritis cases do not have CSF bands and patients with MS tolerate TNF antagonists [7].

The strong association between TNF antagonists and optic neuritis is probably than much more common that reported in the literature. It is, however, a severe complication and the clinical picture should be recognized by those who are treating these patients.

## References

[1] Ten Tusscher MP, Jacobs PJ, Busch MJ, de Graaf L, Diemont WL (2003). Bilateral anterior toxic optic neuropathy and the use of infliximab. *British Medical Journal*.

[2] Chung JH, van Stavern GP, Frohman LP, Turbin RE (2006). Adalimumab-associated optic neuritis. *Journal of the Neurological Sciences*.

[3] von Jagow B, Kohnen T (2008). Anterior optic neuropathy associated with adalimumab. *Ophthalmologica*.

[4] Li SY, Birnbaum AD, Goldstein DA (2010). Optic neuritis associated with adalimumab in the treatment of uveitis. *Ocular Immunology and Inflammation*.

[5] Zannin ME, Martini G, Buscain I (2009). Sudden visual loss in a child with juvenile idiopathic arthritis-related uveitis. *British Journal of Ophthalmology*.

[6] Gupta G, Gelfand JM, Lewis JD (2005). Increased risk for demyelinating diseases in patients with inflammatory bowel disease. *Gastroenterology*.

[7] The Lenercept Multiple Sclerosis Study Group (1999). TNF neutralization in MS: results of a randomized, placebo-controlled multicenter study. *Neurology*.

